# A Multi-Scale Distribution Model for Non-Equilibrium Populations Suggests Resource Limitation in an Endangered Rodent

**DOI:** 10.1371/journal.pone.0106638

**Published:** 2014-09-19

**Authors:** William T. Bean, Robert Stafford, H. Scott Butterfield, Justin S. Brashares

**Affiliations:** 1 Humboldt State University, Arcata, California, United States of America; 2 California Department of Fish and Game, Los Osos, California, United States of America; 3 The Nature Conservancy, San Francisco, California, United States of America; 4 Department of Environmental Science, Policy and Management, University of California, Berkeley, California, United States of America; U.S. Geological Survey, United States of America

## Abstract

Species distributions are known to be limited by biotic and abiotic factors at multiple temporal and spatial scales. Species distribution models, however, frequently assume a population at equilibrium in both time and space. Studies of habitat selection have repeatedly shown the difficulty of estimating resource selection if the scale or extent of analysis is incorrect. Here, we present a multi-step approach to estimate the realized and potential distribution of the endangered giant kangaroo rat. First, we estimate the potential distribution by modeling suitability at a range-wide scale using static bioclimatic variables. We then examine annual changes in extent at a population-level. We define “available” habitat based on the total suitable potential distribution at the range-wide scale. Then, within the available habitat, model changes in population extent driven by multiple measures of resource availability. By modeling distributions for a population with robust estimates of population extent through time, and ecologically relevant predictor variables, we improved the predictive ability of SDMs, as well as revealed an unanticipated relationship between population extent and precipitation at multiple scales. At a range-wide scale, the best model indicated the giant kangaroo rat was limited to areas that received little to no precipitation in the summer months. In contrast, the best model for shorter time scales showed a positive relation with resource abundance, driven by precipitation, in the current and previous year. These results suggest that the distribution of the giant kangaroo rat was limited to the wettest parts of the drier areas within the study region. This multi-step approach reinforces the differing relationship species may have with environmental variables at different scales, provides a novel method for defining “available” habitat in habitat selection studies, and suggests a way to create distribution models at spatial and temporal scales relevant to theoretical and applied ecologists.

## Introduction

Species distribution models (SDMs) have become a cornerstone of theoretical (e.g., [1]) and applied (e.g., [2]) ecological research [3], [4]. In these models, species occurrence data and environmental correlates are used to define the limits of a species distribution ([4]). However, understanding and predicting the relationship between environmental resources and species distributions is complicated by the temporal and spatial scale of analysis, with most SDMs aimed at mapping range-wide associations using abiotic climatic factors. Perhaps because of the broad temporal and spatial scale at which these analyses are conducted, most recommendations suggest that SDMs operate best for populations at equilibrium (e.g.,[5]). By contrast, most species, especially those of conservation concern, are rarely, if ever, at equilibrium [6].

Guisan and Thuiller [7] provide a framework for modeling species distributions at disparate scales. At broad (*e.g.*, biogeographic) spatial and temporal scales, species' distributions tend to be limited primarily by abiotic factors [8]. At finer spatial and temporal scales, species are limited by local community interactions such as resource factors, dispersal, predation, and competition. Guisan and Thuiller's work suggests a multi-step approach to modeling. That is, they encourage practitioners to first define a species' range-wide distribution, and then model limiting factors within that area to better understand relationships with environmental factors at finer spatial or temporal scales. Echoing Hutchinson [9], Guisan and Thuiller [7] refer to the broad-scale, bioclimatic range as the “potential distribution”, and they define the “realized distribution” as the bioclimatic range filtered through dispersal, disturbance, and biotic interactions.

Guisan and Thuiller's [7] research closely parallels work in the field of habitat selection. Johnson [10] defined habitat selection as a strictly hierarchical process, with first-order selection occurring at the level of the physical or geographical range, second-order selection determining the home range, and so on. While Johnson [10] described habitat selection at each scale as a decision-based process by the individual animal, and Guisan and Thuiller [7] formulate it as an environmental filtering process, both clearly suggest that local occurrences are separately constrained within a higher hierarchical biogeographic distribution. As Wiens et al. [11] demonstrated in their landmark study of shrubsteppe birds, not only are these hierarchical levels of habitat selection distinct, animals may select habitat in contrasting directions at different spatial or temporal scales. Environmental factors that predict habitat selection at macro scales (*e.g.* vegetation, cover, temperature, rainfall) may have little predictive value at finer scales, or may even be correlated with selection in opposite directions.

Habitat selection studies have long recognized this problem of temporal or spatial scale incompatibility [12], and resource selection studies frequently examine habitat selection at multiple scales [13]. Despite the long history of research on habitat selection, the problem of defining “available” habitat has been a common and recurring one [14]. Typically, researchers use some measure of a home range, a buffer around used points, or some meaningful political or biological boundary [12]. We suggest that a more appropriate definition of available habitat would follow the well-understood construction of hierarchical habitat selection. That is, a study of habitat selection at multiple scales should follow the theory of Guisan and Thuiller [7] and Johnson [10] by explicitly modeling habitat selection at each hierarchical stage.

Guisan and Thuiller's [7] multi-step approach has been used to model distribution limited by dispersal [15], and habitat type [16], but to our knowledge has not been used to examine the role of resource availability. Resource availability has long been hypothesized as a key factor limiting species' distributions [17], and recent work has supported this (e.g., [18], [19], [20]). In particular, the temporal dynamics of resource availability can be critical to fine-scale distribution modeling in either space or time. While species at broad spatial and temporal scales may be considered at equilibrium, managers are frequently tasked with understanding shifts in distribution at much finer time intervals, such as between years or even seasons [21], [22]. At such temporal scales, variability of resources can greatly impact species distributions, particularly where the presence of a species is positively or negatively related with resource availability [23].

Recent advances in remote sensing techniques have allowed for estimates of resource abundance at fine temporal scales [24]. In particular, the Normalized Difference Vegetation Index (hereafter “NDVI”) has been used as a reliable estimate of biomass in grassland systems [25], and population dynamics in herbivores have been shown to be correlated with NDVI (e.g., [26], [27], [28]). Recent work has shown that NDVI can be a useful predictor of distribution in large herbivores [29].

In this study, we created a multi-step species distribution model for the giant kangaroo rat (*Dipodomys ingens*, hereafter “GKR”). The GKR is an endangered rodent endemic to southern-central California [30]. GKRs are believed to be limited to areas with loamy soils, flat or gently rolling hills, and to areas with mean annual precipitation no greater than approximately 30 cm [31], [32]. First, we estimated the potential distribution (or first-order habitat selection) of the GKR using population-wide occurrence data and static environmental predictor variables (slope, soil particle size, and six climatic variables relating to temperature and precipitation) using the machine-learning method Maxent [33]. Maxent represents an ideal method for modeling the “potential distribution” because it assumes the most uniform distribution of a species' occurrence across the study area, minimally constrained by the provided environmental correlates. Maxent is a presence-background model, and in fact its authors suggest the results may represent the species' potential distribution [33]. Over broader spatial scales and longer time scales, we predicted a negative relationship between GKR presence and precipitation.

We used this model of potential distribution to define available habitat in order to understand finer scale temporal dynamics in GKR population extent (i.e., annual changes in the “realized” distribution). These temporal models incorporated a suite of primary productivity estimates based on the NDVI. In particular, we predicted, based on previous research [30], [32], [34], that GKR presence would show a positive correlation with resource abundance within their potential distribution, possibly with a time lag reflecting a delayed demographic response of GKR to resource availability. Due to the GKR's strong association between population demographics and precipitation, other factors that may also limit population extent (e.g., predation and competition) were not considered in these models.

## Methods

### Study site and focal species

The GKR is a state and federally endangered, burrowing, granivorous rodent endemic to deserts grasslands of California, USA [32]. Once widespread in the western San Joaquin Valley, habitat loss from agriculture and other development have severely restricted its range to a half-dozen populations in and around the California Coast Range [30]. The GKR is considered both a keystone species and an ecosystem engineer [35], [36]. As grasses begin to senesce in April, GKRs remove all herbaceous vegetation from the top of their burrows [31], [37]. This behavior results in clear circles of bare soil, 2–7 m in diameter, where GKRs are present. Aerial surveys have therefore been a useful tool in mapping GKR population extent in years of high primary productivity [38].

This study is primarily focused on the Carrizo Plain National Monument (hereafter “Carrizo”), an area that contains the largest remaining population of GKRs. Carrizo represents the largest representative landscape of San Joaquin Valley annual grassland [39]. Carrizo experiences variable precipitation (mean = 20 cm, sd = 10 cm) that contributes directly to variability in primary productivity, which in turn may drive dramatic annual changes in GKR distribution [32]. Based on aerial surveys, GKR population extent in Carrizo was estimated to expand more than 50% between 2001 and 2006 [38]. Understanding the role of primary productivity in driving these changes is crucial to biodiversity management for this endangered ecosystem. Both the size of the GKR population and its management and monitoring history make Carrizo an ideal study site for examining the role of resource availability on species distributions.

Other factors that often limit a species' realized distribution – predation, parasitism, competition and dispersal – were not believed to be limiting factors for GKR in Carrizo. Within the study area, the open and flat topography, coupled with GKR reproductive habits allow for rapid dispersal. Within Carrizo, GKR appear to be competitively dominant [31], [36]. Because of these features of their ecology, GKR distribution was less likely to be affected by dispersal or competition and, thus, the GKR was a good species for testing models of realized distribution based solely on resource abundance.

This study was carried out in strict accordance with the recommendations in the Guidelines of the American Society of Mammalogists for the Use of Wild Mammals in Research. The protocol was approved by the Animal Care and Use Committee at the University of Californa, Berkeley (R304).

### GKR Distribution

We obtained estimates of GKR distribution from three sources: (1) historical occurrence records from the Global Biodiversity Information Facility (GBIF); (2) contemporary trapping sites throughout GKR range; and (3) aerial surveys of GKR population extent within Carrizo.

Occurrence records were downloaded from the GBIF using the *dismo* package [40] in R [41], and limited to points collected since 1950 (N = 38). We obtained an additional 185 records of GKR presence or absence from trapping conducted in 2010 and 2011. 157 points were selected randomly throughout GKR range, and trapped for three nights with five traps [42]. Eight additional sites were stratified across a range of habitat suitability values from a preliminary distribution model constructed in 2008, and a final 20 presence points were obtained from ongoing trapping in the center of Carrizo [36]. Of the 185 sites trapped, 120 were occupied in either 2010 or 2011, and thus included in the range-wide potential distribution model. Additional details on trapping methodology are provided in Bean et al. [42].

In 2001, 2006, 2010 and 2011, we conducted Carrizo-wide aerial flight surveys in late summer to estimate GKR extent. Using 800 m wide transects with two observers (i.e., monitoring 400 m on each side) and a global positioning system (GPS), we mapped the total extent of active burrows. GPS points were recorded whenever the observers entered or left areas of observable GKR activity. These points were then connected as lines and buffered 400 m on each side to create an estimate of total extent. These surveys were shown to be a reliable estimate of GKR population extent in a given year [38].

### Potential Distribution Modeling

**Figure 1:**
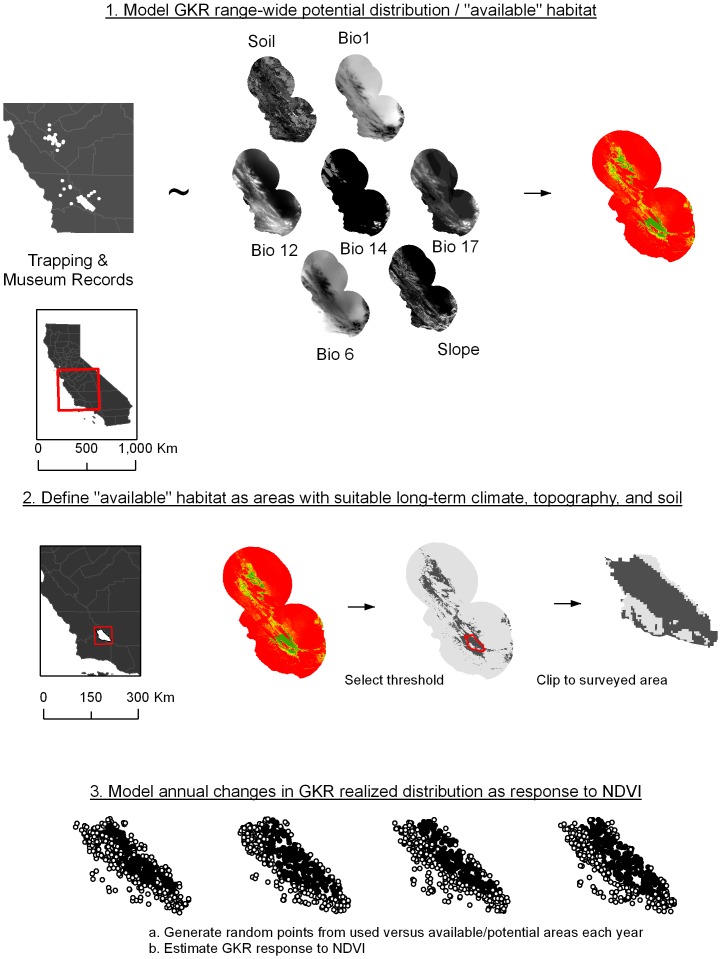
Flow chart of multi-step modeling approach. Here we present a multi-method, multi-scale approach to estimating species distributions. In the first step, Maxent is used to relate contemporary trapping and historical museum records with static environmental variables. The result is a model of potential distribution at a range-wide scale. Predictor variables included soil particle size (“Soil”), annual mean temperature (“Bio1”), minimum temperature of coldest month (“Bio 6”), annual precipitation (“Bio 12”), precipitation of driest month (“Bio 14”), precipitation of driest quarter (“Bio 17”), and slope. We then selected a threshold to define all available habitat for GKR, with the 99% Maxent value for training data used as the threshold. Finally, within the potential habitat in the Carrizo Plain National Monument, we examined annual changes in population extent based on aerial surveys and driven by measures of resource availability (NDVI).

We created a multi-step model to estimate GKR distribution. We first used Maxent to estimate the potential GKR distribution with range-wide occurrence data (museum records and our trapping data] and static environmental variables. Second, we used logistic regression to estimate limits to the potential distribution based on local resource abundance (Fig. 1).

We used the software package Maxent to estimate GKR potential distribution [33]. Maxent uses a maximum entropy approach to estimate the most uniform distribution of a species' occurrence across the study area, minimally constrained by the provided environmental correlates. Maxent is a presence-background model, and therefore may better model the species' potential distribution [33].

To estimate the potential distribution for GKR, we selected a suite of environmental variables believed to limit GKR distribution range-wide. We obtained 19 climate layers [43] frequently used in distribution modeling as independent variables [44]. Bioclim layers are estimated as mean conditions from 1950 to 2000. We limited the variables to six we believed sufficient in describing GKR distribution, and that had limited correlation with each other. These included annual mean temperature (BIO1); annual precipitation (BIO12); minimum temperature of the coldest month (BIO6); precipitation of the driest month (BIO14); and precipitation of the driest quarter (BIO17). In addition, we used slope [45], and soil particle size derived from the SSURGO database [46]. Soil particle size was converted to raster format using ArcGIS 9.2, and all inputs were analyzed at 30 s resolution (the coarsest resolution of all predictor variables). Soil particle size was classified as categorical, with the rest classified continuous.

The output of this initial Maxent distribution model was a map of habitat suitability (Fig. 1), with each 30 s cell representing an index of suitability. To convert the map from a continuous suitability distribution to a binary map of potential distribution, we selected a threshold, above which cells were classified as potential GKR distribution and below which cells were classified as outside potential GKR distribution. A number of methods have been proposed for selecting thresholds [47]–[49]. However, the optimal thresholds recommended in previous work focused on best predicting overall presence or absence for a species. In this case, we were interested in defining the maximum potential distribution for the species. Therefore, in order to err on the side of inclusiveness, we selected a threshold (0.059) that included 99% of presence points from the modeled potential distribution.

### Realized Distribution Modeling

Having produced an estimate of the potential distribution for GKR, we then examined the effects of resource availability on GKR realized distribution for four study years. Because the GKR relies on grass seeds as a food resource, we expected a positive correlation between primary productivity and GKR presence. GKRs dry and cache most of the seeds they collect in underground chambers [37], so GKR presence in a given area may lag primary productivity for a year or more.

To create a spatially explicit measure of primary productivity in Carrizo we acquired 16-day composites (250 m×250 m) of NDVI measured by the Moderate Resolution Imaging Spectroradiometer (“MODIS”) platform [50]. The NDVI is calculated as
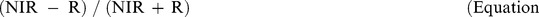
(Equation\;1)where NIR represents spectral reflectance within the near infrared band (841–876 nm), and R represents the visible red band (620–670 nm). Values approaching −1.0 tend to represent areas with water, while areas greater than 0 and approaching 1.0 tend to represent areas of photosynthetic activity [51]. Pre-processed 16-day composites of NDVI measured from MODIS have been shown to better measure primary productivity than single measures. These composites correlate well with biomass in grassland systems [25].

**Figure 2:**
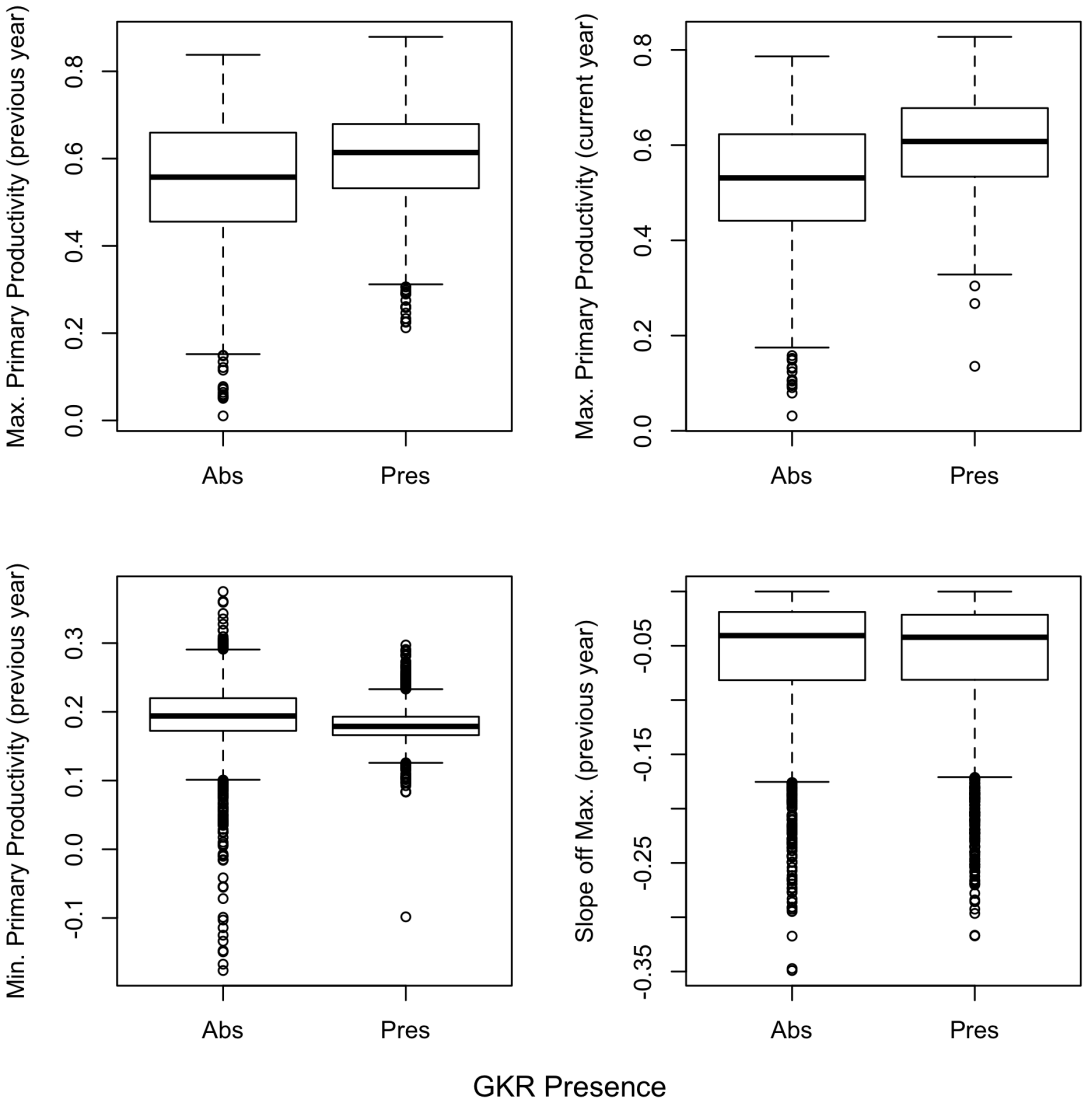
Relationships between primary productivity (measured as NDVI) and GKR presence. GKR were expected to have a positive relationship with maximum primary productivity in the previous and current year; a negative relationship with the minimum primary productivity measured in the previous year; and a negative relationship with the rate of decrease of primary productivity in the previous year. Relationships are shown from 500 random points estimated from aerial surveys in 2011. All differences were significant (t-test, p<0.05).

We created a suite of generalized linear models (GLM) to predict GKR presence using the NDVI for each year (2001, 2006, 2010 and 2011) [13]. We examined two drivers of GKR presence: first, and of primary interest, we tested the effect of primary productivity (*i.e*., resource abundance) on GKR presence. Second, we tested if GKR presence in the previous year would also be a significant predictor of GKR presence in the current year. The independent variables included in the model to evaluate these predictions represented resource abundance in the current or previous year, or were proxies of GKR presence in the previous year (Table 1). These hypotheses were first tested independently before being included in the suite of models (Fig. 2).

**Table 1:** Hypothesized relationships between estimates of primary productivity (NDVI) and the local presence of the giant kangaroo rat.

Candidate Predictors	Hypothesized Mechanism
Maximum NDVI_T1,T0_	Estimate of primary productivity, a bottom-up limitation on GKR presence (with potential one year lag)
Minimum NDVI_T0_	Proxy for GKR presence in previous year
NDVI slope during plant senescence_T0_	Proxy for GKR presence in previous year (GKR remove vegetation more quickly than it senesces)

T1 represents the Normalized Difference Vegetation Index data from the same year as the rat distribution was estimated; T0 is data from the previous year.

First, to estimate resource abundance, we used the highest recorded NDVI value for a given growing season (November through May, the typical rainy season in the Mediterranean climate of coastal California) as an estimate of primary productivity for that 250 m×250 m cell. In estimating distribution for GKR in 2006, for example, we estimated primary productivity in the previous year as the peak NDVI from November to May, 2004–2005; and primary productivity in the current year as peak NDVI from November to May, 2005–2006. NDVI can be inflated by soil moisture if the soil is visible [51] and NDVI appeared to peak approximately 1–2 weeks before the typical peak growth in Carrizo, suggesting that soil moisture was influencing NDVI measurement. However, precipitation and aboveground biomass are correlated and, despite the lag in measurements, peak NDVI has repeatedly been shown to correlate strongly with peak aboveground biomass in grasslands [24], [52].

For three of the four years of surveys, no estimate was available for GKR presence in the previous year. Instead of a direct estimate from aerial surveys, it was therefore necessary to create proxies of GKR presence in the previous year. Because GKR clear their burrow mounds of vegetation, we assumed that GKR would have a direct effect on the NDVI after peak green up. First, we assumed that later in the summer, the areas with GKR would have lower plant biomass than areas without GKR. We therefore included the lowest measured NDVI value from later in the year (April to December) as a proxy for GKR presence, assuming a negative correlation between the two (i.e., areas with GKR would have lower minimum NDVI). Second, we assumed that GKR removed vegetation from around their burrows faster than vegetation naturally senesced. To estimate vegetation removal by GKR, we measured the slope of NDVI decline from its peak. We subtracted the NDVI value from one time step (i.e. 16 days) after peak from the peak NDVI value, and again hypothesized that a larger difference would suggest GKR activity. These two measurements (minimum NDVI and NDVI slope) were used as proxies for GKR presence in an area, and in effect represent a null model of GKR distribution: if current GKR distribution could be predicted solely from the prior year's presence, plant biomass would not be considered a factor limiting the realized GKR distribution.

Although individual GKR burrows (∼27–36 m^2^) represent a small fraction of a single MODIS pixel (250 m×250 m), the heterogeneity of the landscape supports analyses at this scale. The density of the GKR burrows, and the strong difference in signal between the perturbed bare soil on burrow and dried grass off burrow, suggest that a mixed pixel with GKR activity has a significantly different signal than one without GKR activity.

GKR distribution models were ranked using Akaike Information Criteria (AIC) [53]. Models were created for all of the presence points, with year included as a fixed effect. For each year of the model, we used 500 random points from the potential distribution, 250 within GKR realized distribution and 250 outside active areas.

The accuracy of the best model (as identified using AIC) was assessed with the PresenceAbsence package in R [54]. For each model, we calculated a threshold to test predicted presence and absence points for each model. Each threshold was set to the observed prevalence [48]. We then calculated the percent correctly classified (PCC), Cohen's kappa, sensitivity, specificity, and the true skill statistic (TSS, sensitivity + specificity -1), a prevalence-independent measure of accuracy [55]. Testing data was obtained in two ways: for all four years, we randomly selected 500 new points from the aerial surveys in each year, in the same manner as the training data. We also used the set of 105 GKR trapping points in Carrizo collected in 2010 and 2011 to test the models in those years.

## Results

### Potential Distribution Modeling

As expected, GKR potential distribution is limited to a narrow band of habitat on the western San Joaquin Valley and nearby Coast Ranges (Fig. 1). The most important variables in predicting GKR distribution included precipitation of the driest quarter, precipitation of the driest month, and minimum temperature of the coldest month (Table 2). Surprisingly, annual precipitation was not an important predictor of GKR distribution. Instead, precipitation in the driest month and driest quarter were more important predictors. Probability of GKR presence was highest in areas where the driest month received a mean of 0 mm precipitation. Similarly, probability of GKR presence was highest in areas where the driest quarter received a mean of 4 mm precipitation. GKRs inhabited areas that have a narrow band of mean annual temperatures between 14° and 16°C.

**Table 2:** Variable importance for range-wide model of giant kangaroo rat distribution reported by Maxent.

Variable	Percent Contribution	Permutation Importance
Precipitation of Driest Quarter	33.5	29.8
Annual Mean Temperature	21.1	0.4
Precipitation of Driest Month	16.2	33.2
Minimum Temperature of Coldest Month	15.8	22.9
Slope	5.8	5.8
Annual Precipitation	3.9	6.8
Soil Particle Size	3.7	1

**Figure 3:**
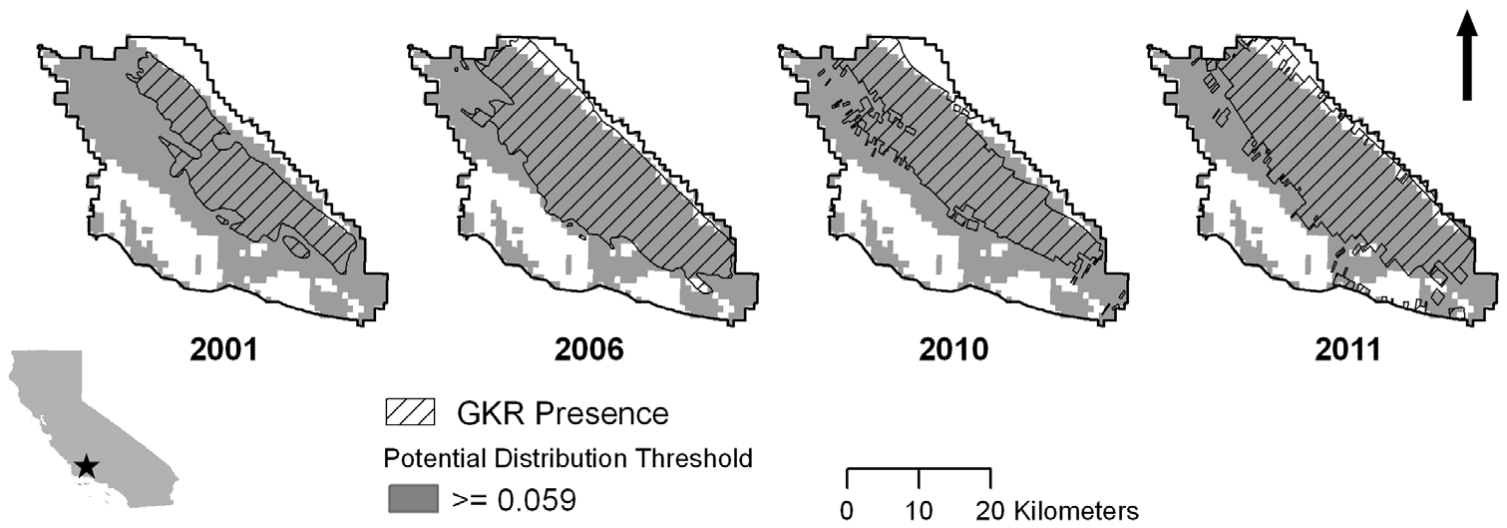
Results of GKR distribution mapping and potential distribution modeling. Hatched polygons show areas of GKR activity in 2001, 2006, 2010 and 2011. Dark grey areas indicate the thresholded potential distribution for GKR from a range-wide Maxent model using presence points from confirmed GKR trapping locations and museum records. A fixed model is unsuitable for predicting annual changes in population extent.

The area classified in the Maxent model as the potential distribution of GKR closely resembled the combined distribution from 2001, 2006, 2010 and 2011 (Fig. 3). However, there were portions of Carrizo in the northwest and southeast classified as suitable that were not part of the realized distribution in any of the years monitored. AUC for the potential distribution model was 0.98.

### Realized Distribution Modeling

In the best model of GKR realized distribution, population extent was positively related to primary productivity in both the previous and current year, suggesting a strong influence of bottom-up regulation on GKR distribution (Table 3). GKR presence in the previous year also was an important predictor of GKR presence in the current year. Both proxies of prior GKR presence performed as expected: GKR distribution was negatively correlated to both minimum NDVI and the slope from the peak NDVI from the previous year. Realized distribution model accuracy from the testing data was “useful” (AUC = 0.74). The threshold was set at 0.50 (as expected, due to the prevalence of the model data [48]). Using the aerial surveys as testing data, model sensitivity was 0.70 and specificity 0.71. The model Kappa score and the true skill statistic (TSS) were 0.40. The best model correctly classified 70.1% of all test points as inside or outside the GKR's estimated realized distribution. Using the trapping data (obtained independently of the training data), sensitivity = 0.65; specificity = 0.66; Kappa = 0.29 and TSS = 0.29, while 65.8% of all test points were correctly classified.

**Table 3:** Logistic regression of GKR presence in relation to NDVI.

Model	AIC	ΔAIC	w_i_
−1.26+6.11*MaxNDVI_T1_+4.15*MaxNDVI_T0_ − 23.79*MinNDVI_T0_ − 3.19*SlopeNDVI_T0_ −0.25*year2006 − 0.55*year2010+0.44*year2011	**2,425.8**	**0**	**1**
MaxNDVI_T1_+MaxNDVI_T0_+MinNDVI_T0_+year	2,439.3	13.50	0
MaxNDVI_T1_+MinNDVI_T0_+SlopeNDVI_T0_+year	2,452.7	26.91	0
MaxNDVI_T1_+MinNDVI_T0_+year	2,461.5	35.72	0
MaxNDVI_T0_+MinNDVI_T0_+SlopeNDVI_T0_+year	2,502.9	77.05	0
MaxNDVI_T0_+MinNDVI_T0_+year	2,514.7	88.94	0
MaxNDVI_T1_+year	2,623.9	198.15	0
MaxNDVI_T1_+SlopeNDVI_T0_+year	2,624.8	199.03	0
MaxNDVI_T1_+MaxNDVI_T0_+year	2,625.2	199.35	0
MaxNDVI_T1_+MaxNDVI_T0_+SlopeNDVI_T0_+year	2,626.1	200.34	0
MaxNDVI_T0_+year	2,702.4	276.56	0
MaxNDVI_T0_+SlopeNDVI_T0_+year	2,703.6	277.82	0

The full model performed the strongest. In this model, GKR presence is positively correlated with peak primary productivity in the current year and previous year, and negatively correlated with minimum NDVI and NDVI slope in the previous year. This suggests that the best predictor of GKR presence in a given year is a positive correlation with resource abundance over two years, and presence in the area the previous year.

## Discussion

This study joins a growing body of literature that attempts to use ecological theory on limits to population extent and species ranges to inform, interpret and advance species distribution models (e.g. [1], [16], [56], [57]). Specifically, we presented a technique of multi-step modeling to define a species' potential and realized distribution, and in doing so explored the relationship between primary productivity and animal distribution.

Consistent with theory on potential and realized distributions [4], [7], our results showed that the potential distribution of GKR was larger than any of the distributions observed in the four years of aerial surveys. In other words, there were areas within Carrizo that should have been suitable for GKR, but monitoring documented them as uninhabited. This result supports conclusions of Guisan and Thuiller [7], and Grinnell [17] and Hutchinson [9] before them, who suggested that species' distributions are limited by more than fixed environmental conditions, a fundamentally important concept for distribution modelers and ecologists.

The fact that distribution models built only on static bioclimatic factors may poorly estimate realized distributions has several important implications for how these models are applied to questions in biodiversity conservation. For example, distribution models are often relied upon to project the impact of climate change on species' distributions. Without incorporating mechanisms that limit the focal species' realized distribution, these models are likely over-estimating the range of conditions within which the species will survive and reproduce under different climate change scenarios [58],[59]. At the same time, we may be ignoring important local ecological processes by examining patterns of distribution at range-wide spatial scales [60]. In this study, GKR potential distribution was limited to areas with little to no rain in the driest months of the year; therefore, future increases in precipitation might be expected to reduce GKR distribution. However, we found that within the area of potential distribution, GKR were positively correlated with primary productivity. It is possible, then, that an increase in mean annual precipitation would decrease the potential distribution range-wide (where agriculture has already rendered suitable habitat uninhabitable), but increase the realized distribution within a core conservation area.

Within the potential distribution, we found that the best model of factors limiting the realized distribution of GKR showed a clear, positive correlation between primary productivity (measured as peak NDVI) and the presence of GKR in the previous year. This result conforms to recent findings from studies of other species that show rapid changes in distribution in response to temporal and spatial variability of NDVI (*e.g*., Mongolian gazelles (*Procapra gutturosa*; [19], [20]); and African buffalo (*Syncerus caffer*; [61], [62]).

A key step in our approach required a selection of threshold to convert a continuous model of habitat suitability at the range-wide scale to a binary presence-absence map of potential distribution (or “available habitat” *sensu* Johnson [1980]). An alternative approach that might prove useful to explore would utilize the continuous distribution of habitat suitability as an informative prior in modeling habitat selection at finer scales. However, it is unclear whether animals select habitat in this manner. We suggest that, for GKRs, climate, soil, and topography serve as a simple filter to defining the potential distribution. That is, for example, either a GKR can construct a burrow in a particular soil type or it can't – we do not expect GKRs to have a continuous response to resource abundance in relation to soil particle size. Nevertheless, additional research on the relationship across habitat selection at multiple scales is warranted.

This study of GKR distribution in Carrizo, while conducted at a relatively small spatial scale, focused on the temporal dynamics of species' distributions. Niche and distribution theory tend to assume a species is at equilibrium, but this study and others (e.g., [21], [63]) show that for many applications, considering the temporal dynamics of a species' distribution is essential. Although the importance of non-static suitability models in grassland systems has been recognized [64], the difficulties in addressing such variability have thus far limited research in this area [65].

This study focused specifically on resource abundance as a limiting factor for GKR.

While the approach presented here combining distribution models at different scales allows new insights, it is not without its shortcomings. One particular problem is our inability to identify the “true” potential distribution. By its very nature, it may be impossible to know a species' potential distribution; in fact the potential distribution may only be a theoretical construct. We can only measure the realized distribution and estimate the potential distribution from those measurements. This issue is highlighted regularly in the invasive species modeling literature. Species that appear to have a limited distribution in their native range often show a spectacular ability to live in “unsuitable” conditions when introduced to new areas (e.g., [66], [67]). In these cases, the species' realized distribution in its native range is so limited by competition, dispersal, and other ecological factors that any estimate of its potential distribution will be woefully inadequate for predicting the spread of a species. Oftentimes, ecological limits to the realized distribution may be correlated with environmental conditions, thereby preventing true knowledge of the species' limits of its potential distribution. In this case, additional steps (*e.g*. physiological tests) may be required to estimate its potential distribution.

As for GKR's competitive dominance, the relationship between precipitation limitation and competition may be impossible to untangle. The *Heteromyidae* in general appear to have evolved to claim a desert-grassland niche unfilled by other small mammals. The observed relationship between dry summer months and GKR presence may be as much related to the *lower* limit for larger rodents (*e.g*., the California ground squirrel, *Otospermophilus beecheyi*) than an upper limit for GKR. Again, this illustrates the conceptual difficulty surrounding niche theory, but the temporal mechanisms outlined in this study ought to remain relevant. GKR display differing responses to precipitation at range-wide and local scales. This fact is a crucial finding for those interested in modeling ecologically relevant species' distributions.

Incorporating detailed mechanisms into species distribution models, at ecologically relevant scales and informed by ecological theory is an important next step in the field of spatial ecology. We have presented an approach to estimating a species' potential distribution and address questions about the ecological limits to its realized distribution. We presented further evidence that non-equilibrium populations are often limited not just by fixed, environmental conditions, but also other ecological conditions that vary spatially and temporally. Such research will be important as distribution modeling moves from the “how” to the “why.”
